# Primary Mucinous Adenocarcinoma of the Urinary Bladder with Signet-Ring Cells: Description of an Uncommon Case and Critical Points in Its Management

**DOI:** 10.1155/2016/6080859

**Published:** 2016-12-18

**Authors:** Fabrizio Di Maida, Giuliano Amorim Aita, Daniele Amorim Aita

**Affiliations:** ^1^Section of Urology, Department of Surgical, Oncological and Stomatological Sciences, University of Palermo, Palermo, Italy; ^2^Section of Urology, Hospital Universitário da Universidade Federal do Piauì, Teresina, PI, Brazil; ^3^Section of Oncology, Hospital Universitário da Universidade Federal do Piauì, Teresina, PI, Brazil

## Abstract

We present an uncommon case of mucinous adenocarcinoma of the bladder (MAB) with signet-ring cells extensively infiltrating prostate gland and pelvic/retroperitoneal lymph node stations and not responsive to usual systemic chemotherapy regimens. This case highlights the important features of MAB including the pattern of tumor spread, the tendency for initial misdiagnosis, and the importance of immunohistochemical study in order to define its primary origin from the bladder and choose the most appropriate treatment since the beginning.

## 1. Introduction

Primary adenocarcinoma of the urinary bladder is a rare urologic neoplasia, accounting for less than 2% of all malignant bladder tumors [1]. Mucinous and signet-ring cells histological variants are even rarer [2, 3]. Primary adenocarcinoma of the bladder is characterized by a very aggressive behavior and poorly responding to radio or chemotherapy at first-line treatment. This tumor has overlapping histological characteristics with adenocarcinomas arising from other primary sites such as colon, prostate, and gynecological tract, whereby immunohistochemistry is essential to determine the primary origin from the bladder. Because of its rarity and the difficulties in locating the primary tumor, site diagnosis is often delayed, so that palliative chemotherapy is the only possible therapy for almost 50% of patients.

## 2. Case Presentation

A 57-year-old man suffering from hypertension and chronic kidney disease presented low abdomen pain and occasional palpable mass in the hypogastrium. He mostly complained of obstructive LUTS such as frequent pain or stiffness in pelvic area, hesitancy, slow and weak urine stream, straining to urinate, and overflow incontinence. Digital rectal examination revealed an enlarged prostate but no prostate nodules. PSA was equal to 0.37 ng/mL. Ultrasonography (US) of abdomen and pelvis showed the presence of considerable chronic urinary retention with a postvoid residual (PVR) equal to almost 200 cc and confirmed the presence of an enlarged prostate with neither hypoechoic nor suspicious lesions. Moreover a bladder thickening with mild hydronephrosis was evident, linked in the first instance to benign prostatic hyperplasia (BPH).

Judging by the current the patient underwent transurethral resection of prostate (TURP). Subsequent histopathological examination showed a prostatic adenocarcinoma Gleason score of 10 (5 + 5) involving 90% of tissue resected. On physical examination, the patient reached Eastern Cooperative Oncology Group (ECOG) performance status of two. There was no evidence of peripheral lymphadenopathy or visceromegaly.

**Figure 1:**
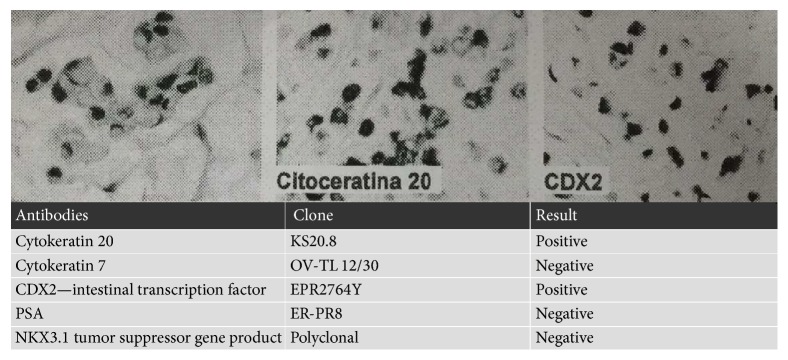
Immunohistochemical examination showing positivity for CK20 and CDX2 and negativity for CK7, PSA, and NKX3.1.

Hypointense areas on T2-weighted gadolinium-enhanced magnetic resonance imaging (MRI) of the abdomen and pelvis were reported in the apex on the left side. The base of the prostate gland showed a marked contrast enhancement bilaterally. A single lymph node of the internal iliac chain showed an irregular appearance measuring 1.3 cm. A moderate wall thickening of the bladder and a mild dilatation of the proximal portion of the ureters bilaterally were also evident. Radical prostatectomy plus lymphadenectomy were performed one month later. Histopathological examination confirmed the diagnosis of mucinous adenocarcinoma with signet-ring cells. The tumor had already infiltrated the prostatic capsule and the margins of resection were positive. As mentioned above, it is usually difficult to define the primary origin of the tumor, so it was necessary to perform an immunohistochemical study. The tumor was found to be positive for CK20, CDX2, and EMA but negative for PSA, NKX3.1, and CK7 (Figure 1). This immunohistochemical panel was not suggestive of primary adenocarcinoma of the prostate but could correspond either to a primary vesical or a colonic adenocarcinoma.

**Figure 2:**
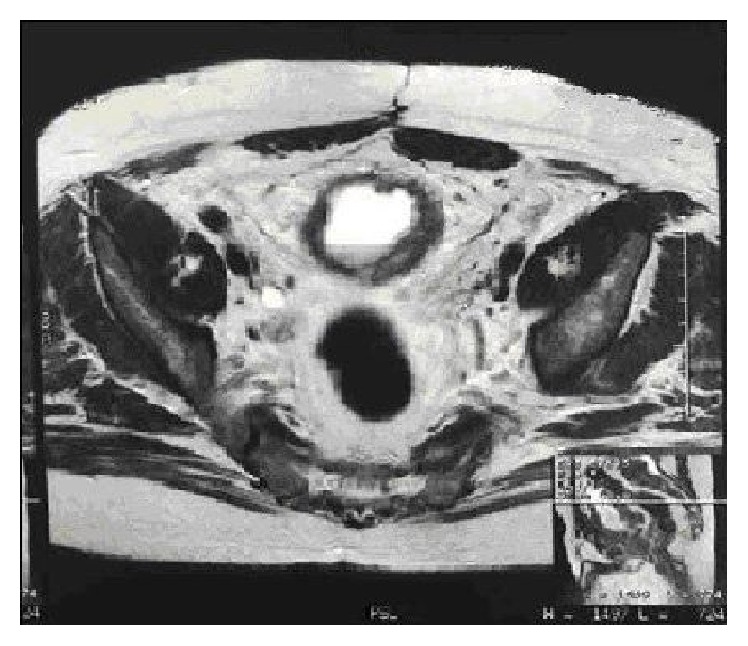
MRI scan section: the MRI scan revealed vesical thickened walls, more evident in the lower third of the bladder, associated with an exophytic lesion.

A second MRI of the abdomen and pelvis showed retroperitoneal, iliac, and aortic-iliac lymph nodes increased in volume. Moreover the MRI confirmed the bladder thickening, more evident in the lower third of the bladder where it was associated with an exophytic lesion (Figure 2). The patient underwent a vesical biopsy and histopathological examination confirmed the diagnosis of primary MAB with signet-ring cells, grading G3. As the patient presented in advanced stage, a palliative-intent 6-cycle chemotherapy with gemcitabine and Taxol was started and two months later the patient returned to evaluate the effectiveness of the treatment. He complained about pelvic discomfort, dysuria, and urgency. A TC/PET showed FDG captation by inguinal lymph node stations. A new salvage chemotherapy regimen with FOLFOX was about to be administered, but patient's poor general conditions forced postponing the treatment. Two months later the patient was hospitalized due to a severe urinary tract infection, probably related to the progression of the disease, and succumbed to the tumor within the next 3 weeks.

## 3. Discussion

Primary adenocarcinoma of the urinary bladder is a rare malignancy, characterized by a wide range of histological varieties, such as colonic type, mucinous type, signet-ring cell type, clear cell type, hepatoid, and adenocarcinoma not otherwise specified [1]. All these histological varieties are commonly grouped as urachal and nonurachal forms. Adenocarcinoma arising from urachal remnants is the most common form of vesical adenocarcinoma, accounting for 22% to 35% of all vesical adenocarcinomas. It usually has a male predominance and tends to occur in the vesical dome or anterior wall [4]. In case of nonurachal adenocarcinoma, many authors suggest that this tumor arises through a process of intestinal metaplasia stimulated by chronic irritation. We had to deal with a nonurachal primary MAB with signet-ring cells.

Since the first MRI showed a moderate bladder thickening and no exophytic lesions, this radiological finding was initially related to the effect of chronic urinary retention. Immunohistochemistry was essential to locate the primary origin of the tumor and exclude other adenocarcinomas, above all prostatic and colonic metastatic adenocarcinomas. An immunohistochemical panel positive for CK20, CDX2, and EMA but negative for PSA, NKX3.1, and CK7 was not suggestive of primary adenocarcinoma of the prostate but could correspond either to a primary vesical or a colonic adenocarcinoma.

Vesical adenocarcinoma is usually positive for CEA, CDX-2, MUC-1, MUC-2, and MUC-3, as well as metastatic colonic adenocarcinoma, and positive for both CK7 and CK20. On the contrary the typical colonic adenocarcinoma staining profile is CK7 negative and CK20 positive [5]. In our case it was a primary MAB histologically similar to colonic adenocarcinoma and consequently showing a CK7 negative staining pattern unlike other adenocarcinomas of the bladder. These variants may further delay the diagnosis. Only by integrating clinical data, radiological workup, and immunohistochemical study it is possible to reach a high diagnostic accuracy since the beginning.

Moreover even when the immunohistochemical study is correctly performed, due to the rarity of this tumor and the tendency for initial misdiagnosis, unfortunately at the time of diagnosis about 46% of patients already have stage IV tumor (including lymph node positivity, T4b stage, and distant metastases) [6] requiring the use of palliative chemotherapy. In case of an operable tumor a radical cystectomy represent the gold standard treatment.

Few data is available on adjuvant chemotherapy and results are still controversial. Romics et al. [7] report good response using adjuvant chemotherapy with cisplatin and 5-fluorouracil. Akamatsu et al. [6] indicate tumor stage and increased carcinoembryonic antigen levels as significant prognostic factors. In our case metastatic disease required palliative chemotherapy, so gemcitabine plus Taxol were administered as first-line treatment, but the patient showed no clinical response. Since MAB shows a histologic similarity to colonic cancer, some authors recently suggested that chemotherapy regimens usually used for gastrointestinal tumors could be considered as alternative schemes of treatment. In the literature there are only three cases describing the use of FOLFOX in primary metastatic adenocarcinoma of the bladder. Tatli et al. [8] and Tran and McKendrick [9] reported complete response. Teo et al. [10] suggested adding bevacizumab to FOLFOX, obtaining a sustained response for ten months. If these results will be confirmed FOLFOX could represent a real possibility for a second-line chemotherapy in metastatic vesical adenocarcinoma. Unfortunately in our case the patient died before the second-line treatment could be administered. This suggests the need for an early diagnosis when the patient's performance status still permits the administration of polychemotherapy regimens burdened by great toxicity.

## 4. Conclusions

Primary adenocarcinoma of the bladder is a very uncommon malignancy. A comprehensive evaluation of the gastrointestinal and gynecological tracts by an immunohistochemical analysis should be early performed to exclude adenocarcinomas arising from other primary sites and choose the most appropriate therapy. Gold standard treatment for nonurachal adenocarcinoma of the bladder is radical cystectomy. The addition of adjuvant chemotherapy might ameliorate long-term survival in case of early cystectomy. Recent evidences have shown that an oxaliplatin plus fluoropirimidine regime (FOLFOX) should be considered in case of failure of first-line chemotherapy treatment. Further studies are needed.
